# First report of a patient meeting criteria for both multisystem inflammatory syndrome in children and adult onset Still’s disease

**DOI:** 10.1186/s41927-022-00320-9

**Published:** 2022-12-28

**Authors:** Maya Alexandri, Julisa Patel, Eli Paul, Lynne W. Coule

**Affiliations:** 1 Emergency Medicine, Medical College of Georgia, Augusta University Medical Center, 1120 15th Street, Augusta, GA 30912 USA; 2 Pediatric Rheumatology, Medical College of Georgia, Children’s Hospital of Georgia, 1446 Harper Street, Augusta, GA 30912 USA; 3 University of Colorado at Boulder, Boulder, CO 80309 USA; 4 Pediatric Emergency Medicine, Medical College of Georgia, Children’s Hospital of Georgia, 1446 Harper Street, Augusta, GA 30912 USA

**Keywords:** Multisystem inflammatory syndrome in children, MIS-C, Novel coronavirus disease 2019, COVID-19, Systemic juvenile idiopathic arthritis, SJIA, Adult onset Still’s disease, AOSD, Still’s disease, Macrophage activation syndrome, MAS, Case report

## Abstract

**Background:**

COVID-19 is associated with a postinfectious hyperinflammatory disorder, multisystem inflammatory syndrome in children (MIS-C), that shares characteristics with still’s disease, known as systemic juvenile idiopathic arthritis (SJIA) in children younger than 16, and adult onset Still’s disease (AOSD) in children 16 and older. Both MIS-C and SJIA/AOSD can be complicated by macrophage activation syndrome (MAS), a potentially fatal condition of cytokine storm.

**Case presentation:**

We present a 16 year-old male who developed quotidian fever, headache, conjunctival injection, sore throat, nausea and vomiting, diarrhea, rash, and symmetrical polyarticular arthralgia/arthritis 4 weeks after exposure to SARS-CoV-2 and 2 weeks after his first vaccination against COVID-19. Our patient’s laboratory results were significant for elevated inflammatory markers and acute phase reactants. He met criteria for diagnosis with both MIS-C and AOSD. After receiving first-line treatment for both diseases, IVIG and methylprednisolone, our patient improved.

**Conclusion:**

MAS is a life-threatening rheumatological emergency, and physicians must be able to identify diseases, like MIS-C and AOSD, that may be complicated by MAS. Our patient’s distinguishing feature on presentation was symmetrical polyarticular arthralgia/arthritis, which has not been associated with MIS-C. Simultaneously, AOSD—which is associated with polyarticular arthralgia/arthritis—is only now being recognized as a possible post-infectious entity in the aftermath of COVID-19 infection. In patients like our own, who meet criteria for both MIS-C and AOSD, administering first line treatment for both diseases may be best practice.

## Background

Since April 2020, the novel coronavirus disease 2019 (COVID-19), caused by severe acute respiratory syndrome coronavirus 2 (SARS-CoV-2), has been recognized to have rare, severe sequelae in children. Termed multisystem inflammatory syndrome in children (MIS-C), this post-infectious hyperinflammatory disorder—like COVID-19 itself—has a wide array of presentations [1]. The World Health Organization, Centers for Disease Control, Royal College of Paediatrics and Child Health, and American College of Rheumatology have all proposed varying case definitions [1–4].

MIS-C was initially likened to Kawasaki Disease (KD), a rare pediatric systemic vasculitis that usually involves medium-sized vessels, particularly coronary arteries [1, 5]. Among other reasons, the two can share the following presenting symptoms and features: fever, bilateral conjunctivitis, mucocutaneous involvement, edematous hands and feet, cardiac pathology, and elevated inflammatory markers [5].

Parallels also exist between MIS-C and another rheumatologic entity: systemic juvenile idiopathic arthritis (SJIA), also known as Still’s Disease and—in children 16 years or older—as adult onset Still’s disease (AOSD). Fever, rash, arthralgia/arthritis, cardiac pathology, and elevated inflammatory markers are common to both MIS-C and SJIA/AOSD [5–7].

Like MIS-C, AOSD can be complicated by macrophage activation syndrome (MAS), a potentially fatal state characterized by dysregulated activation of T and B cells leading to excessive production of pro-inflammatory cytokines [8, 9]. This “cytokine storm” eventually can lead to end organ damage if not treated and carries a high mortality rate [8, 9].

According to the American College of Rheumatology, diagnosing MIS-C is appropriate when patients meet the following criteria: (1) unremitting fever greater than 38 °C, (2) epidemiological link to COVID-19, and (3) at least two or more of the following: rash, gastrointestinal symptoms, edema of hands and feet, oral mucosal changes, conjunctivitis, lymphadenopathy, or neurological symptoms [1]. Although arthritis is not a defining symptom of MIS-C, one study found that arthritis was a clinical feature in 9.1% of the cases of MIS-C included in the review [5].

Like MIS-C, AOSD is a clinical diagnosis and one of exclusion, after consideration of alternative diagnoses, including infections, malignancy, and other inflammatory diseases [1, 3, 10]. Multiple diagnostic criteria have been proposed. Using the Fautrel criteria, which has been validated with a sensitivity of 87% and a specificity of 97.8% [11–13], a diagnosis of AOSD is warranted if the patient meets four major criteria or three major criteria and two minor criteria. The major criteria are: spiking fever ≥ 39 °C, arthralgia, transient erythema, pharyngitis, neutrophil polymorphonuclear proportion ≥ 80%, glycosylated ferritin proportion ≤ 20%. The minor criteria are: maculopapular rash and leukocytosis ≥ 10,000. Fautrel’s standard has no exclusion criteria [12]. The Yamaguchi criteria has also been validated for AOSD with a sensitivity of 96.2% and a specificity of 92.1%, but it has exclusion criteria that reduce its clinical utility, as the Yamaguchi criteria apply only after extensive work up [10, 12]. Per Yamaguchi, diagnosis is appropriate if a patient meets five or more criteria, at least two of which are major [10]. Its major criteria are: fever of ≥ 39 °C for a week or more; arthralgia of 2 weeks or longer; maculopapular, salmon-pink rash; leukocytosis ≥ 10,000 with a neutrophil polymorphonuclear proportion ≥ 80% [10, 12]. The minor Yamaguchi criteria are: pharyngitis or sore throat, lymphadenopathy and/or splenomegaly, liver enzyme abnormalities, negative for rheumatoid factor or antinuclear antibodies [10, 12]. As many as 86–100% of patients with AOSD have arthralgia/arthritis as a clinical feature, especially involving the wrists, knees, and ankles [14].

Diagnosis has implications for, among other aspects, treatment. Patients diagnosed with MIS-C receive IVIG and aspirin; if patients are in shock, suffer organ-threatening injury, or are refractory to IVIG, a moderate consensus in the American College of Rheumatology recommends low-to-moderate dose glucocorticoids as adjunctive therapy [1]. By contrast, patients diagnose with AOSD are treated first line with steroids; second line treatment is methotrexate, and refractory cases are treated with IL-1, IL-6, and TNF inhibitors [9, 14]. Patients with AOSD are not treated with aspirin.

We present the first case, to our knowledge, of a patient who met criteria for both MIS-C and AOSD.

## Case presentation

Our patient was a 16 year-old Caucasian male presenting to the Emergency Department at the Children’s Hospital of Georgia (CHOG) with quotidian fever, intermittent headache, bilateral conjunctival injection, sore throat, nausea and vomiting, diarrhea, polyarticular arthritis, and rash. About a month prior to presentation, patient had had cold-like symptoms. His mother had concomitantly had similar symptoms, though worse in severity, and had tested positive for COVID-19. Patient was not tested for COVID-19 at that time. Two weeks prior to presentation, patient had received his first vaccination against SARS-CoV-2 with the Pfizer/BioNTech vaccine.

Symptom onset was a week prior to presentation. Patient’s symptoms began with fever (at least once per day), headache, and sore throat. He also had episodes of nausea, vomiting, and diarrhea that resolved 3 days into his course of illness.

Patient was evaluated at an urgent care, where he was administered a rapid strep test and a Monospot; he tested negative for both. He was nonetheless discharged with amoxicillin for empiric treatment of strep pharyngitis and ondansetron for symptomatic relief of his nausea.

Four days into his illness, patient’s oral intake decreased. His symptoms persisted, and he additionally felt weak. He presented at his local emergency department, where he was assessed, but no changes were made to his management.

Thereafter, patient developed bilateral joint edema, erythema, heat, and pain in his metacarpophalanges, elbows, knees, and ankles. The joint pain impaired patient’s ability to walk. In addition, patient developed a rash.

Patient saw his pediatrician, who found that patient’s erythrocyte sedimentation rate and C-reactive protein were elevated four times above the normal limit. The pediatrician discontinued patient’s amoxicillin and recommended that patient be seen at a children’s hospital with pediatric rheumatology and infectious disease capacity.

Patient presented in the CHOG Emergency Department the next day. At that time, his joint edema, pain, and erythema had improved, although he had persistent erythema over his metacarpophalanges joints and edema bilaterally in his ankles. His primary complaint was sore throat, which was 7 out of 10 in severity and improved with administration of ketorolac. His vital signs were: temperature 36.7 °C, heart rate 87, respiratory rate 18, blood pressure 113/73, SpO_2_ 100%.

On physical examination, patient had bilateral conjunctival injection and pharyngeal erythema without exudates. This skin over his metacarpophalangeal joints was erythematous, and he had 1+, non-pitting edema bilaterally at the ankles. A blanching, salmon-pink, maculopapular rash was apparent at the suprapubic area and descended bilaterally along both legs to his ankles. Patient’s abdominal examination was negative for hepatosplenomegaly.

Patient’s laboratory results were significant for D-dimer elevated to 311, ESR elevated to 60, fibrinogen elevated to 777, CRP elevated to 21.7, ALT elevated to 115, neutrophils elevated to 84, and eosinophils elevated to 7. He was also lymphopenic at 5. (See Table 1 for laboratory results.) Patient’s platelets and non-glycosolated ferritin were within normal limits. A respiratory viral panel was negative, and patient tested negative for COVID-19. He was admitted for workup.

**Table 1:** Patient’s laboratory results upon presentation

White blood cells	8.9 thous/mm^3^
Platelets	207 thous/mm^3^
Neutrophils	84% (H)
Lymphocytes	5% (L)
Eosinophils	7% (H)
ESR	60 mm/h (H)
ALT	115 u/L (H)
D-dimer	311 ng/mL (H)
Fibrinogen	777 mg/dL (H)
CRP	21.7 mg/dL (H)
LDH	190 u/L
Aldolase	15.1 u/L (H)
CK	29 u/L (L)
Troponin	< 0.010 ng/mL

In the course of patient’s inpatient care, he was found to be positive both for SARS-CoV-2 antibodies and for antibodies formed after vaccination. Although he had an elevated aldolase at 15.1, his CK was low at 29. Patient’s LDH was within normal limits. Glycosylated ferritin, antinuclear antibody, RH factor, anticitrullinated protein, and ANCA were not tested.

Patient was diagnosed with MIS-C, meeting the criteria set forth by the American College of Rheumatology of fever, rash, oral mucosal involvement, bilateral conjunctivitis, and previous infection with COVID-19.

He could also have been diagnosed with AOSD. Patient met the Fautrel criteria with spiking fevers, arthralgia/arthritis, transient erythema, pharyngitis, neutrophil polymorphonuclear proportion ≥ 80%, and salmon-pink, maculopapular rash. Applying the Yamaguchi criteria is not appropriate because patient was not fully worked up for the exclusion criteria, owing to the low index of suspicion for malignancy, infection, or other inflammatory disease. Patient nonetheless met the following four Yamaguchi criteria, including two major criteria: once-a-day fever lasting a week, salmon-pink maculopapular rash, pharyngitis, and liver enzyme abnormalities. Although five Yamaguchi criteria are required for diagnosis, patient’s arthalgia/arthritis had not lasted 2 weeks at the time of presentation, and he received treatment before his symptoms were allowed to endure for that duration. Additionally, patient was not tested for rheumatoid factor or antinuclear antibodies.

An echocardiogram performed inpatient was normal.

Patient’s treatment comprised IVIG and diphenhydramine, methylprednisolone, and aspirin. He had an episode of hives in response to IVIG that was treated with diphenhydramine.

He thus received the first line treatments both for MIS-C and AOSD. (In accordance with the American College of Rheumatology recommendations, methylprednisolone is indicated for patients in shock or who are refractory to IVIG [1]. Our patient was hemodynamically stable on presentation and throughout his treatment, and he appeared to respond to IVIG.) His symptoms improved, and he was discharged after 3 days.

Patient has continued to improve since his discharge. Patient took oral steroids for 4 weeks following discharge. He has required additional NSAIDS for sore throat and joint pain relief following the taper of the steroids, and he has experienced some skin peeling on his fingers. He experienced no recurrence following completion of the steroid course. His 2-week follow-up examination with cardiology was unremarkable, as was his 2 month repeat echocardiogram. Patient did not experience adverse or unexpected events in the course of his treatment. (For a timeline of patient’s disease course, please see Table 2.

**Table 2:** Timeline of patient’s disease process

About 4 weeks prior to presentation	Patient developed cold-like symptoms. His mother had similar symptoms, though worse in severity, and she test positive for COVID-19
About 2 weeks prior to presentation	Patient was vaccinated against COVID-19 with Pfizer/BioNTech vaccine
One week prior to presentation	Patient developed fever, headache, sore throat, nausea, vomiting, and diarrhea
About 4 days prior to presentation	Patient’s GI symptoms resolved. Patient tested negative on rapid strep and Monospot tests. Patient was prescribed amoxicillin and ondansetron
Three days prior to presentation	Patient’s PO intake decreased. He was evaluated at an outside hospital Emergency Department, and no changes were made to his management
Two days prior to presentation	Patient developed bilateral joint edema, erythema, heat, and pain in his metacarpophalanges, elbows, knees, and ankles. Additionally, he develops a rash
One day before presentation	Patient’s pediatrician evaluated patient and found elevated CRP and ESR. Patient’s pediatrician discontinued patient’s amoxicillin and recommended that patient be seen at a children’s hospital with rheumatology and infectious disease capacity
Day of presentation	Patient’s primary complaint was sore throat. His joint edema, pain, and erythema had improved, despite persistent erythema over his metacarpophalanges joints and edema bilaterally in his ankles. Patient was admitted to the hospital
One day after presentation	Patient was evaluated by rheumatology and started on IVIG, methylprednisolone, and aspirin
Three days after presentation	Patient had a negative echocardiogram. He was discharged from the hospital on a steroid taper
About 2 weeks after discharge	At follow up with rheumatology, patient had a normal joint exam and some skin peeling. He had been taking NSAIDs for relief of sore throat and joint pain that worsened with the steroid taper. At follow up with cardiology, patient had a negative echocardiogram. He continued on aspirin
About 2 months after discharge	Patient had a negative echocardiogram

Consent was obtained for this case report.

## Discussion and conclusions

Polyarticular arthralgia/arthritis is a distinctive feature of a disease presentation. A symmetric polyarticular arthralgia/arthritis narrows the differential to varieties of inflammatory arthritis, like rheumatoid arthritis, systemic lupus erythematosus, vasculitis, AOSD, and infectious arthritis [14–16]. Our patient’s presentation supports the supposition that polyarticular arthralgia/arthritis may also be a clinical feature of MIS-C. It concomitantly bolsters the literature documenting AOSD arising in the aftermath of COVID-19 infection or vaccination [6, 7, 17, 18].

Based on our review, scant literature documents arthralgia/arthritis as a feature of MIS-C. Indeed, Bagri et al. describe arthritis as a “rare” clinical feature in their cohort of 31 MIS-C patients: only one patient (3% of their cohort) had arthritis, described as a bilateral knee arthritis [19]. Yener et al., studied a larger cohort of 154 children diagnosed with MIS-C and identified arthritis as a clinical feature in 9.1% of them, but did not classify the arthritis as mono- or polyarticular [4]. Our patient contributes to this literature by providing an example of a case meeting criteria for MIS-C that also features polyarticular arthritis of the metacarpophalanges, elbows, knees, and ankles.

Our literature review also uncovered two reports of patients being diagnosed with AOSD after having contracted COVID-19 [6, 7]. The first report involved a 29 year-old woman who, 6 months after having had COVID-19, presented with fever, sore throat, salmon-colored rash, arthralgia, leukocytosis, and cardiac involvement [7]. Her symptoms were refractory to prednisolone, but they resolved with administration of anakinra, an IL-1 receptor antagonist that is a treatment for AOSD refractory to first line steroid treatment.

The second report was of a 27 year-old man who, 8 weeks after a case of COVID-19, presented with fever, sore throat, polyarthralgia, rash, and leukocytosis with neutrophilia [6]. This patient’s symptoms resolved with treatment with methylprednisolone.

Our patient contributes a third example to this literature. Viral infection has been proposed as an etiology of AOSD [14, 16]. The literature includes evidence that rubella, measles, echovirus 7, coxsackievirus B4, cytomegalovirus, and Epstein–Barr viruses may all be implicated in AOSD [14, 20]. The example of our patient, as well as that of the two previous case reports, suggests that COVID-19 should be added to the list of triggering viruses.

Intriguingly, our patient’s case raises an additional possibility. His disease onset was 4 weeks after his infection with COVID-19, but 2 weeks after his first vaccination shot against SARS-CoV-2. Our review of the literature found two reports of patients being diagnosed with AOSD after having been vaccinated against SARS-CoV-2 [17, 18]. Magliulo et al. report AOSD being diagnosed in a 45 year-old female, with symptom onset 5 days following her second shot of the Moderna mRNA-1273 vaccine [18]. Leone et al. report development of AOSD in a 36 year-old male, with symptom onset the day after his first dose of the AstraZeneca ChAdOx1 nCoV-19 vaccine [17]. In both cases, the patients’ symptoms were refractory to steroids, but resolved after administration of anakinra. Although parsing whether our patient’s symptom presentation was provoked by his illness with COVID-19, his vaccination with the Pfizer/BioNTech BNT162b2 vaccine, or yet another etiology is not possible, our patient brings the number of documented cases of AOSD following partial or full vaccination against SARS-CoV-2 to three, and is the first to involve the Pfizer/BioNTech vaccine.

Our patient is the first reported case meeting criteria both for MIS-C and AOSD and illustrates the importance of maintaining a broad differential for diagnosing patients presenting with rheumatological symptoms after infection with COVID-19. The landscape of possible illnesses post-COVID is still being mapped. Although research is ongoing to identify biomarkers that might distinguish MIS-C and AOSD [8, 9, 14], the diagnosis of both entities is clinical, and many of the biomarkers under investigation are non-routine and of limited availability to clinicians.

Based on experience, the presence of hepatosplenomegaly may weigh more in favor of AOSD. By contrast, cardiac abnormalities may be more indicative of MIS-C. Pronounced inflammation that rapidly resolves may be more consistent with MIS-C. Once treatment has begun, non-relapse after steroid taper is more suggestive of MIS-C.

At stake in the diagnosis is treatment. In both MIS-C and AOSD, intervention may avert or abort cytokine storm, a potentially lethal state that is theorized to have MAS as a common pathophysiological process [8]. In addition, MIS-C patients may benefit from aspirin for coronary artery protection [1]. Treatment with both IVIG and steroids, even for patients who are not in shock, as was done with our patient, administers first line therapy for MIS-C and AOSD. This course, along with administration of aspirin, may be best practice where available, when a patient meets diagnostic criteria for both entities.

## Data Availability

Not applicable.

## Abbreviations

AOSD: Adult onset Still’s disease
CHOG: Children’s Hospital of Georgia
COVID-19: Novel coronavirus disease 2019
KD: Kawasaki disease
MAS: Macrophage activation syndrome
MIS-C: Multisystem inflammatory syndrome in children
SARS-CoV-2: Severe acute respiratory syndrome coronavirus 2
SJIA: Systemic juvenile idiopathic arthritis

## References

[1] Henderson LA, Canna SW, Friedman KG, Gorelik M, Lapidus SK, Bassiri H, Behrens EM, Ferris A, Kernan KF, Schulert GS (2021). American College of Rheumatology Clinical Guidance for multisystem inflammatory syndrome in children associated with SARS–CoV-2 and hyperinflammation in pediatric COVID-19: version 2. Arthritis Rheumatol.

[2] World Health Organization. Multisystem inflammatory syndrome in children and adolescents with COVID-19. May 2020. www.who.int/publications/i/item/multisystem-inflammatory-syndrome-in-children-and-adolescents-with-covid-19.

[3] Centers for Disease Control and Prevention. Emergency preparedness and response: health alert network. May 2020. https://emergency.cdc.gov/han/2020/han00432.asp.

[4] Royal College of Paediatrics and Child Health. Guidance: paediatric multisystem inflammatory syndrome temporally associated with COVID-19. June 2020. https://www.rcpch.ac.uk/resources/guidance-paediatric-multisystem-inflammatory-syndrome-temporally-associated-covid-19-pims.

[5] Otar Yener G, Paç Kısaarslan A, Ulu K (2021). Differences and similarities of multisystem inflammatory syndrome in children, Kawasaki disease and macrophage activating syndrome due to systemic juvenile idiopathic arthritis: a comparative study. Rheumatol Int.

[6] Alshablan A, Jabbad R, Tayeb S, Albeshri T, Yelamanchili SR, Rabie N, Almutairi F, Samannodi M (2021). Diagnosis of adult onset Still’s disease in a patient who has recovered from coronavirus-19. Clin Med Insights Case Rep.

[7] Bamidis AD, Koehler P, di Cristanziano V, Rasche K, Demirel B, Bacher P, Hallek M, Kochanek M, Klein F, Hofmann SC, Wesselmann U, Kofler DM (2021). First manifestation of adult-onset Still's disease after COVID-19. Lancet Rheumatol.

[8] Rodriguez-Smith JJ, Verweyen EL, Clay GM, Esteban YM, de Loizaga SR, Baker EJ, Do T, Dhakal S, Lang SM, Grom AA, Grier D, Schulert GS (2021). Inflammatory biomarkers in COVID-19-associated multisystem inflammatory syndrome in children, Kawasaki disease, and macrophage activation syndrome: a cohort study. Lancet Rheumatol.

[9] Aydın F, Çelikel E, Ekici Tekin Z, Coşkun S, Sezer M, Karagöl C, Kaplan MM, Tekgöz N, Kurt T, Özcan S, Kavurt AV, Özkaya Parlakay A, Çelikel Acar B (2021). Comparison of baseline laboratory findings of macrophage activation syndrome complicating systemic juvenile idiopathic arthritis and multisystem inflammatory syndrome in children. Int J Rheum Dis.

[10] Yamaguchi M (1992). Preliminary criteria for classification of adult Still’s disease. J Rheumatol.

[11] Fautrel B, Zing E, Golmard JL, Le Moel G, Bissery A, Rioux C (2002). Proposal for a new set of classification criteria for adult-onset still disease. Medicine (Baltim.).

[12] Feist E, Mitrovic S, Fautrel B (2018). Mechanisms, biomarkers and targets for adult-onset Still’s disease. Nat Rev Rheumatol.

[13] Lebrun D (2018). Validation of the Fautrel classification criteria for adult-onset Still’s disease. Semin. Arthritis Rheum.

[14] Giacomelli R, Ruscitti P, Shoenfeld Y (2018). A comprehensive review on adult onset Still’s disease. J Autoimmun.

[15] Pujalte GG, Albano-Aluquin SA (2015). Differential diagnosis of polyarticular arthritis. Am Fam Physician.

[16] Bhargava J, Panginikkod S. Still Disease. [Updated 2021 Jul 10]. In: StatPearls [Internet]. Treasure Island (FL): StatPearls Publishing; 2021 Jan-. https://www.ncbi.nlm.nih.gov/books/NBK538345/.

[17] Leone F, Cerasuolo PG, Bosello SL, Verardi L, Fiori E, Cocciolillo F, Merlino B, Zoli A, D’Agostino MA (2021). Adult-onset Still’s disease following COVID-19 vaccination. Lancet Rheumatol.

[18] Magliulo D, Narayan S, Ue F, Boulougoura A, Badlissi F (2021). Adult-onset Still’s disease after mRNA COVID-19 vaccine. Lancet Rheumatol.

[19] Bagri NK, Deepak RK, Meena S (2021). Outcomes of multisystem inflammatory syndrome in children temporally related to COVID-19: a longitudinal study [published online ahead of print, 2021 Oct 19]. Rheumatol Int.

[20] Wouters JM, van der Veen J, van de Putte LB (1988). Adult onset Still’s disease and viral infections. Ann Rheum Dis.

